# The Impact of Synbiotics on the Bacterial Flora During the Course of Chronic Sinusitis

**DOI:** 10.3390/medicina61071306

**Published:** 2025-07-20

**Authors:** Karolina Goroszkiewicz, Grażyna Lisowska, Grażyna Stryjewska-Makuch, Olga Karłowska-Bijak, Maciej Misiołek

**Affiliations:** 1 Department of Laryngology and Laryngological Oncology, Leszek Giec Upper-Silesian Medical Centre of the Silesian Medical University, 40-635 Katowice, Poland; 2 Department of Otorhinolaryngology and Laryngological Oncology in Zabrze, Medical University of Silesia, 40-055 Katowice, Poland

**Keywords:** chronic rhinosinusitis, synbiotics, probiotics, retrospective studies

## Abstract

*Background and objectives*: Chronic rhinosinusitis (CRS) is a multifactorial inflammatory condition often associated with microbiome imbalance (dysbiosis). Recent studies highlight the potential role of synbiotics—combinations of probiotics and prebiotics—in modulating the microbiota and supporting immune responses. The authors of this study aimed to evaluate the impact of oral synbiotic supplementation on the sinus microbiota in patients undergoing endoscopic sinus surgery (ESS) for CRS. *Materials and Methods*: A total of 425 adult patients with CRS were enrolled in a multicenter retrospective study. According to EPOS 2020 guidelines, participants qualified for ESS. The intervention group (*n* = 194) received a synbiotic preparation for 6–8 weeks before and after surgery; the control group (*n* = 231) received no supplementation. Intraoperative and follow-up bacteriological samples were collected and analyzed. Statistical analysis included chi-square, t-tests, Wilcoxon tests, and ANOVA models. *Results*: Patients receiving synbiotics showed a significant reduction in pathogenic bacterial colonies postoperatively compared to the control group. In the synbiotic group coagulase-negative staphylococci appeared more frequently. Patients in the synbiotic group required significantly less postoperative antibiotic therapy (*p* < 0.05). Both groups exhibited an increase in Gram-positive and physiological flora and a decrease in Gram-negative bacteria following ESS. *Conclusions*: Synbiotic supplementation may beneficially influence the composition of the sinus microbiota and reduce pathogenic bacterial colonization following ESS. The findings suggest that synbiotics could serve as a supportive strategy in CRS treatment, potentially decreasing the need for postoperative antibiotics.

## 1. Introduction

Chronic rhinosinusitis (CRS) is a common condition with significant clinical relevance. It is estimated to affect 5.5–28% of the population; however, when objective diagnostic criteria are applied, the prevalence drops to 1.7–8.8% in the United States and 10.9% in Europe [1]. These differences are partly due to environmental exposures and tobacco smoking. CRS is associated with a considerable economic burden: direct costs (including pharmacological and surgical treatments) amount to USD 10–13 billion annually, while indirect costs (such as productivity loss) exceed USD 20 billion [2].

In recent years, there has been growing interest in the role of the microbiome in the pathophysiology of chronic diseases, including CRS [3]. The microbiota of mucosal surfaces and the skin plays a crucial role in immune regulation, and its disturbances—even during the prenatal period—can influence the development of various diseases [3].

The nasal and sinus microbiome provides a protective function by maintaining immune homeostasis. In healthy individuals, the microbial composition is diverse, with *Corynebacterium* and *Staphylococcus* being predominant [4]. Although *Staphylococcus* can act as a pathogen, in this context it functions as a commensal, cooperating with *Corynebacterium*. The latter has been shown to inhibit pathogenic *Streptococcus* through the production of fatty acids [4,5].

Other bacterial genera present in healthy sinuses include *Moraxella*, *Streptococcus*, and *Haemophilus*—also acting as commensals. International studies have found no significant differences in the nasal microbiome among healthy individuals across different continents [4].

The disruption of microbiome balance, known as dysbiosis, can be triggered by genetic factors, diet, medications, and lifestyle [6]. Dysbiosis can affect various organ systems and may be diagnosed by analyzing microbial metabolites. An increasing number of studies suggest that microbiome manipulation may become a therapeutic target [6].

In the context of CRS, studies have shown an increased presence of *Streptococcus*, *Moraxella*, and *Haemophilus* [7]. Although the core sinus microbiome is shared between healthy and diseased individuals, those with CRS with nasal polyps (CRSwNP) show an increase in *Streptococcus* and a decrease in *Corynebacterium*, which may indicate dysbiosis [4].

Koeller et al. demonstrated that more pronounced microbiome changes occur in CRS without nasal polyps (CRSsNP), where *Flavobacterium*, *Pseudomonas*, *Pedobacter*, *Porphyromonas*, *Stenotrophomonas*, and *Brevundimonas* were more frequently observed [8]. Other researchers have also reported differences between groups—for example, *Staphylococcus*, *Alloiococcus*, and *Corynebacterium* are more common in patients with nasal polyps, while *Streptococcus*, *Haemophilus*, and *Fusobacterium* are more prevalent in those without polyps [9,10].

According to the World Health Organization (WHO), probiotics are live microorganisms that, when administered in adequate amounts, confer a health benefit on the host. In contrast, prebiotics are non-digestible substances that selectively stimulate the growth and/or activity of specific beneficial microorganisms in the gut, thereby contributing to the host’s health. The combination of both—referred to as synbiotics—is designed to enhance the survival and implantation of probiotic strains by providing a supportive substrate.

Probiotics exhibit antiviral activity and influence the microbiome, immune modulation, and epithelial barrier function [11,12,13]. Key characteristics of probiotics include the ability to adhere to the nasal epithelium, compete with pathogens, and adapt to environmental conditions (e.g., oxygen levels and pH) [13].

Studies have shown the beneficial effects of probiotics in asthma treatment, including the reduction of Th2 and Th17 responses, improvements in skin and mucosal barrier function, and activation of anti-inflammatory mechanisms such as the PPAR-γ pathway [14]. Reductions in IL-4, IL-6, and IL-17 levels, improvement in lung function parameters, and an increase in regulatory T cells have also been observed [15,16,17]. Probiotic supplementation may improve asthma control and enhance patients’ quality of life [18].

In the context of CRS and allergic rhinitis (AR), probiotics also show promising effects. Although they do not always lead to clear symptom improvement, they may reduce the risk of relapse and improve quality of life [13,19,20,21].

In summary, disturbances in the sinus microbiome may play a significant role in the pathogenesis of CRS, and variations in its composition may influence disease subtypes. Manipulating the microbiota, including the use of probiotics, is an increasingly attractive therapeutic strategy that warrants further investigation.

The aim of the study was to evaluate the effect of synbiotics on the bacterial flora of the sinuses in patients with chronic rhinosinusitis undergoing endoscopic surgery.

## 2. Materials and Methods

### 2.1. Study Population

The study included patients with chronic rhinosinusitis (CRS) who qualified for endoscopic sinus surgery (ESS) in accordance with the EPOS 2020 (European Position Paper on Rhinosinusitis and Nasal Polyps 2020) guidelines [22].

The study was conducted in two centers: the Department of Otorhinolaryngology and Oncological Laryngology at the Upper Silesian Medical Center Prof. Leszek Giec of the Medical University of Silesia in Katowice, Poland and the Department of Otorhinolaryngology and Oncological Laryngology in Zabrze, Poland.

A total of 425 patients aged 19 to 84 years were enrolled, with a mean age of 48.9 years. Among them, 194 patients received a synbiotic supplement, while 231 patients formed the control group without synbiotic supplementation. The study population consisted of 56% men and 44% women. All patients were recruited between 1 January 2022, and 28 February 2023.

Inclusion criteria:

- Diagnosis of CRS according to EPOS 2020 guidelines
- Qualification for endoscopic sinus surgery

Exclusion criteria:

- Secondary CRS according to EPOS 2020
- Benign and malignant neoplasms of the sinuses
- Immunosuppressive treatment
- Age under 18 years
- Lack of qualification for general anesthesia
- Mental or physical condition preventing completion of questionnaires
- Pregnancy
- Unilateral lesions
- Infection within 6 weeks prior to surgery
- Antibiotic therapy within 6 weeks prior to surgery
- Surgery performed during the pollen season in patients allergic to pollen or mold spores

Data from 498 patients were reviewed, of whom 425 met the inclusion criteria and were enrolled in the study. As this was a retrospective study, the ability to control for potential sources of bias was inherently limited. However, efforts were made to minimize variability between groups by applying consistent inclusion and exclusion criteria across all patients.

A completed STROBE checklist for retrospective case–control studies has been submitted as Supplementary Material to ensure transparency and comprehensive reporting of the study design and findings.

### 2.2. Methodology

Prior to study initiation, the Bioethics Committee of the Medical University of Silesia in Katowice, Poland was consulted regarding the need for ethical approval. A written confirmation was received stating that approval was not required (reference number: PCN/CBN/0022/KB/244/21).

Patients in the study group received a synbiotic preparation in a dose of one capsule per day for a period of 6–8 weeks before and 6–8 weeks after ESS. The symbiotic (Multilac, USP Zdrowie) composition included the following:

- Probiotics: *Lactobacillus acidophilus, Lactobacillus casei, Lactobacillus plantarum, Lactobacillus rhamnosus, Lactobacillus delbrueckii subsp. bulgaricus, Bifidobacterium bifidum, Bifidobacterium breve, Bifidobacterium longum, Streptococcus thermophilus*
- Prebiotic: Fructooligosaccharides

As part of the surgical qualification process, each patient was advised to undergo dental and allergological consultations, including allergy testing. All patients, after initial qualification and prior to ESS, were required to use intranasal corticosteroids at a dose of 200 μg/24 h for a minimum of 12 weeks. Upon hospital admission, medical history was obtained, and an ENT examination was performed. Routine perioperative antibiotic prophylaxis was not used. Between postoperative days 7 and 10, patients attended a follow-up visit in the hospital outpatient clinic, where the need for antibiotic treatment was evaluated. Antibiotic therapy was initiated only in cases with clinical signs of infection, such as purulent nasal discharge observed during endoscopic examination, headache, and fever. Patients also completed the SNOT-22 (Sino-Nasal Outcome Test) questionnaire and VAS (Visual Analog Scale) assessments (separately for nasal discharge, nasal obstruction, headache, and olfactory disturbances), both before the procedure and 6–8 weeks after surgery.

### 2.3. Bacteriological Procedures

Intraoperative swabs were collected from the middle nasal meatus during ESS. Prior to surgery, the external nose and nasal vestibule were cleansed and disinfected by threefold wiping with a sterile gauze soaked in Octenisept. The material was collected using a swab with AMIES transport medium and delivered to the laboratory. Follow-up samples were collected at postoperative control visits in the outpatient clinic 6–8 weeks after ESS using the same procedure.

### 2.4. Laboratory Procedures

Bacteriological analysis included identification and quantification of bacterial strains present in the samples. For aerobic culture, the following media were used: Columbia blood agar (Gram-positive bacteria), MacConkey agar (Gram-negative bacteria), Sabouraud agar (fungi), and chocolate agar under increased CO_2_ conditions (for Neisseria and Haemophilus species). Anaerobic bacteria were cultured on Schaedler agar. All cultures were incubated at 35–37 °C for 24–48 h, except for fungal cultures and Schaedler agar, which were incubated for seven days. During incubation, the remaining material on the swab was stored in enrichment medium (brain–heart infusion broth) for seven days. Identification and antimicrobial susceptibility testing were performed using the Vitek 2 Compact system.

A comprehensive review of the scientific literature was carried out as part of the study preparation. Literature searches were conducted in three major databases: PubMed, Medline, and Science Direct. Publications on the sinus microbiome, treatment of chronic rhinosinusitis, and the influence of synbiotics on bacterial flora were analyzed. A critical review of the available studies allowed for the identification of a research gap and the formulation of research hypotheses that served as the foundation for the present study.

### 2.5. Statistical Analysis

Statistical analysis was performed using R Studio (R 4.4.2) with the R programming language. Descriptive statistics were presented in tabular form. The normality of variable distribution was assessed using histograms and QQ plots in R Studio.

Associations between variables were evaluated using the chi-square test for categorical variables, the Student’s t-test for continuous variables with approximately normal distributions, and the Wilcoxon test for non-normally distributed continuous variables. Comparisons before and after surgery, as well as between the synbiotic and control groups, were conducted using ANOVA models for normally distributed variables, generalized estimating equations (GEE) for non-normally distributed variables, and chi-square tests for categorical data. A *p*-value < 0.05 was considered statistically significant.

## 3. Results

The study group consisted of 425 individuals, 46% of whom used synbiotics. Men constituted the majority of the participants, accounting for 56% of the group. The average age of the subjects was 49 years, with a standard deviation of 15 years. The mean duration of the disease was 6.5 years. The study analyzed a number of quantitative variables, which are presented in Table 1.

Nasal polyps (the clinical phenotype of CRSwNP) were present in 42% of the patients. The most commonly reported symptoms included nasal discharge (86% of participants) and nasal obstruction (88%), as well as headaches (63%) and olfactory disturbances (56%). Additionally, 61% of patients had never undergone sinus surgery before, while 24% had one previous surgery and 15% had undergone more than one.

An analysis of qualitative variables was performed, including gender, reported symptoms, number of previous sinus surgeries, and comorbidities. It was found that patients who used synbiotics were significantly less likely to require antibiotic therapy after surgery. The data are presented in Table 2.

Quantitative variables such as age, disease duration, and bacterial colony counts were also analyzed. Patients who used synbiotics had a significantly lower bacterial colony count in postoperative nasal swabs compared to those who did not use synbiotics (Table 3).

Further analysis compared bacteriological results between patients using and not using synbiotics. In the study group, coagulase-negative Staphylococcus spp. were cultured significantly more often both before and after surgery than in the control group (Table 4).

Among the 231 patients who did not use synbiotics, 84 received antibiotic therapy following surgery at the outpatient clinic. In contrast, 61 patients from the synbiotic group received antibiotics. The decision to initiate antibiotic therapy was made by the attending physician based on the patient’s clinical condition. The specific antibiotic was selected according to the results of the bacteriological culture.

An analysis of bacteriological results in patients who did not receive antibiotics showed significantly lower prevalence of *E. coli, Staphylococcus aureus*, and *Staphylococcus aureus MSSA* in the synbiotic group (Table 5).

Bacteriological outcomes were compared in patients using and not using synbiotics, both before and after surgery (Table 6). A significant decrease in E. coli colonies was observed postoperatively in both groups. Additionally, an increase in the presence of physiological flora was noted in both groups after surgery. In the control group, there was a significant postoperative reduction in Klebsiella oxytoca and Staphylococcus epidermidis. In the synbiotic group, the number of Klebsiella pneumoniae colonies decreased significantly after surgery.

In both groups, a significant increase in the proportion of Gram-positive bacteria was observed postoperatively, accompanied by a decrease in the proportion of Gram-negative bacteria cultured from swabs.

Subsequently, bacteriological results before and after ESS were compared specifically among patients who did not receive antibiotic therapy postoperatively (Table 7). Among these patients, an increase in the proportion of physiological flora was observed after surgery in both the control and study groups.

## 4. Discussion

The primary objective of the undertaken study was to determine whether the administration of synbiotics to patients with chronic rhinosinusitis (CRS) prior to endoscopic sinus surgery (ESS) influences the sinus microbiome. Observations indicated that patients who used synbiotics had a significantly lower count of pathogenic bacterial colonies in nasal swabs collected postoperatively compared to those in the control group. Similar findings were reported by Gluck et al. [23], who observed a significant reduction in the presence of potentially pathogenic bacteria in the nasal cavity among individuals consuming a probiotic beverage, particularly concerning Gram-positive bacteria. The intake of probiotics reduced nasal colonization by pathogenic bacteria such as *Staphylococcus aureus* and *Streptococcus pneumoniae* [23].

To date, results from two clinical trials have been published assessing the systemic impact of oral probiotics in CRS. In a multicenter, double-blind, placebo-controlled study, the efficacy of *Enterococcus faecalis* in reducing acute exacerbations in CRS patients was evaluated. The authors noted a reduction in the number of symptomatic CRS episodes among patients who received a 6-month oral course of *E. faecalis*, with benefits persisting for 8 months post-treatment [24]. Another study assessed the efficacy of oral probiotics in the form of chewable tablets containing 500 million active cells of the *L. rhamnosus* strain administered over 8 weeks. However, the results did not demonstrate significant benefits of probiotic treatment compared to placebo [25].

In recent years, most studies have focused on the topical application of probiotic preparations to the nasal cavity and sinuses, such as sprays or irrigations. In the study by Mårtensson et al., the effect of the “LAB” spray (containing *Lactobacillus* and *Bifidobacterium*) on patients with CRS with nasal polyps was examined. The authors found that the probiotic spray was well tolerated and safe to use but did not observe a significant impact on disease progression. No differences in the nasal microbiome were noted between the “LAB” and placebo groups, neither for commensal bacteria nor for CRS pathogens after treatment with “LAB” and placebo [26].

In the study by Endam et al., *Lactococcus lactis* W136 was administered nasally in the form of irrigation to CRS patients, demonstrating safety and a significantly beneficial effect on patient-reported symptoms. Microbiome changes associated with the treatment were limited to an increase in the number of *Dolosigranulum pigrum* colonies, a bacterium considered potentially beneficial in the upper respiratory tract [27]. For several years, the *Lactococcus lactis* W136 preparation has been available on the market in the USA and Canada in the form of a nasal and sinus irrigation powder. According to a study published by de Boeck et al., *L. casei AMBR2* may inhibit the growth of *M. catarrhalis, H. influenzae*, and *S. aureus*, exhibiting antimicrobial activity [28].

Analyzing the relationship between synbiotic intake and postoperative outcomes in patients undergoing ESS, it was found that in both groups—those taking synbiotics and those not taking them—a reduction in the number of pathogenic bacterial colonies in swabs after ESS was observed compared to the preoperative state. This may indicate the effectiveness of surgical intervention in reducing the number of pathogenic bacterial colonies in the sinuses and nasal cavity.

Additionally, the impact of ESS on the composition of the sinus microbiota was observed. In the group of patients taking synbiotics, *coagulase-negative Staphylococci* were cultured significantly more frequently both before and after ESS, which may suggest an influence of synbiotics on the composition of the bacterial microflora. In both studied groups, after ESS, there was a significant decrease in the number of cultured E. coli, an increase in the proportion of physiological flora, a decrease in the percentage of Gram-negative bacteria, and an increase in the percentage of Gram-positive bacteria. Furthermore, in the group of patients using synbiotics, a decrease in the number of cultured *Klebsiella pneumoniae* was observed after ESS, while in the control group, a decrease in the number of cultured *Klebsiella oxytoca* and *Staphylococcus epidermidis* was noted. Among patients using synbiotics who did not require antibiotic therapy postoperatively, significantly less frequent occurrences of *E. coli, Staphylococcus aureus*, and *Staphylococcus aureus MSSA* were observed.

In the conducted study, the composition of the bacterial flora of the patients after the procedure was influenced not only by synbiotic intake but also by the ESS procedure itself. As previously mentioned, published studies indicate that ESS can lead to changes in the composition of the sinus microbiome, including a reduction in the number of MRSA strains and changes in bacterial diversity and dominance [29,30,31].

In summary, the conducted studies found that the use of synbiotics in patients after ESS significantly reduces the number of pathogenic bacterial colonies compared to the control group. These results suggest that both surgical interventions and synbiotic preparations can effectively reduce bacterial load in the sinuses and nasal cavity. The analysis also showed that synbiotics may specifically influence the composition of the bacterial microflora, manifested by a more frequent presence of *coagulase-negative Staphylococci* and a decrease in the number of *Klebsiella pneumoniae* after the procedure.

Additionally, patients using synbiotics without the need for antibiotic therapy less frequently exhibited the presence of bacteria such as *E. coli* or *Staphylococcus aureus*. These results are consistent with previous studies indicating a reduction in nasal colonization by pathogenic bacteria after probiotic consumption. Clinical trials have also assessed the efficacy of oral probiotics in reducing recurrences of chronic sinusitis, with mixed results regarding their effectiveness.

Comparing postoperative treatment outcomes between patients taking synbiotics and those not taking them, with particular attention to indications for antibiotic therapy after surgery, it was observed that patients undergoing ESS who took synbiotics as recommended for 6 weeks before and 6 weeks after surgery required antibiotic therapy significantly less frequently in the early postoperative period. The obtained results may suggest a beneficial effect of synbiotics on the patients’ immune system.

Probiotics, which are a key component of synbiotics, may support the immune system by promoting a balance between Th1 and Th2 responses. This is important for regulating allergic reactions and inflammatory states [32].

The action of probiotics on the gut microbiome also plays a key role in their beneficial health effects. Probiotic supplementation has shown promising results in reducing the need for antibiotic treatment in adult patients by maintaining microbiological diversity, reducing the abundance of antibiotic resistance genes, and preventing antibiotic-associated diarrhea [33]. Studies indicate that probiotics can help maintain the composition of the gut microbiome, prevent disturbances caused by antibiotics, and potentially reduce the risk of infectious diseases, thereby reducing the need for antibiotics [33,34]. Probiotics have demonstrated beneficial effects on the host organism, potentially reducing the risk of upper respiratory tract infections and, consequently, the need for antibiotic treatment in adult patients [35]. The results of these studies underline the need for further standardized clinical trials to more precisely investigate the potential of probiotics in reducing the need for antibiotic treatment in adult patients [34].

In the context of ESS, these results suggest that synbiotic supplementation may support both the immune system and the sinonasal microbiome, resulting in fewer postoperative complications and a reduced need for antibiotics. This is a significant finding that may contribute to optimizing postoperative management and improving clinical outcomes in patients undergoing ESS. However, it is important to acknowledge that our study was retrospective, and the results should be interpreted with caution. Prospective, randomized controlled trials with a double-blind design are needed to confirm these findings and fully assess the clinical impact of synbiotic supplementation in the pre- and postoperative care of ESS patients.

## 5. Conclusions

Synbiotics have a beneficial effect on the composition of the bacterial microflora following endoscopic sinus surgery. Their use significantly reduces the number of pathogenic bacterial colonies while increasing the presence of coagulase-negative staphylococci. Synbiotic supplementation supports the nasal and sinus microbiome, contributing to a reduced incidence of postoperative complications. Moreover, patients receiving synbiotics require antibiotic therapy significantly less often after surgery, indicating a positive impact on the healing process and a reduction in postoperative infections. Based on our findings, we recommend considering the routine use of synbiotic supplementation as part of pre- and postoperative care in patients undergoing endoscopic sinus surgery. The observed benefits suggest that synbiotics may support the recovery process and help maintain a balanced sinonasal microbiome. These results warrant further investigation but may already inform modifications to clinical practice protocols aimed at improving surgical outcomes and minimizing antibiotic use.

## Figures and Tables

**Table 1:** Descriptive statistics of quantitative variables.

Variable	*n*	Min	Max	Median	Q1	Q3	Mean	SD
Age	425	19	84	47	37	61	48.878	14.928
Duration of symptoms (years)	344	0	50	4	2	10	6.464	7.058
Lund–Mackay score	364	1	24	11	8	16	11.742	5.39
VAS nasal discharge before ESS	356	0	10	5.25	4	8	5.625	2.764
VAS nasal obstruction before ESS	356	0	10	7	4	8.5	6.296	2.758
VAS headache before ESS	356	0	10	4	1	7	4.048	3.254
VAS smell before ESS	356	0	10	4	0.5	9	4.688	3.988
SNOT-22 score before ESS	353	0	108	46	30	60	45.742	19.811
VAS nasal discharge after ESS	297	0	10	3	1	5	3.458	2.618
VAS nasal obstruction after ESS	297	0	10	2	0	5	2.798	2.766
VAS headache after ESS	297	0	10	1	0	4	2.392	2.717
VAS smell after ESS	297	0	10	2	0	7	3.621	3.632
SNOT-22 score after ESS	295	0	85	24	12	40.5	28.112	19.995

**Table 2:** Comparison of qualitative variables between the group of patients using synbiotics and the group not using synbiotics.

Variable	No Synbiotic *n* = 231 (100%) ^1^	Synbiotic *n* = 194 (100%) ^1^	*p* ^2^
Sex			0.003
Male	144 (62%)	92 (47%)	
Female	87 (38%)	102 (53%)	
Presence of nasal discharge	195 (86%)	167 (86%)	>0.99
Nasal obstruction	198 (87%)	171 (88%)	0.89
Headaches	129 (57%)	135 (75%)	0.008
Olfactory disturbances	130 (57%)	106 (55%)	0.66
Previous sinus surgeries:			0.58
None	132 (58%)	126 (65%)	
One	58 (25%)	43 (22%)	
More than one	38 (17%)	25 (13%)	
Hypertension	72 (31%)	53 (27%)	0.43
Diabetes mellitus	9 (4%)	10 (5%)	0.70
Asthma	51 (22%)	46 (24%)	0.80
Inhalant allergy	70 (30%)	66 (34%)	0.49
NSAID hypersensitivity	28(12%)	20 (10%)	0.65
Smoking	64 (28%)	44 (23%)	0.27
Occupational exposure to harmful agents	36 (16%)	38 (20%)	0.35
Postoperative antibiotic use	84 (51%)	61 (36%)	0.018

^1^ n (%); ^2^ Pearson’s chi-squared test.

**Table 3:** Comparison of quantitative variables between the group of patients using synbiotics and the group not using synbiotics.

Variable	Group	*n*	Min	Max	Median	Q1	Q3	Mean	SD	*p*
Age	Without synbiotic	231	21	80	48	38.5	62.5	49.792	14.592	0.17
	Synbiotic	194	19	84	46	35	60	47.789	15.286	
Duration of symptoms (in years)	Without synbiotic	152	0	40	4	2	8.25	6.131	6.739	0.428
	Synbiotic	192	0.25	50	5	2	10	6.727	7.308	
Bacterial colony count before ESS	Without synbiotic	227	0	4	2	1	3	1.89	1.245	0.58
	Synbiotic	192	0	3	3	0	3	1.922	1.318	
Bacterial colony count after ESS	Without synbiotic	181	0	4	1	0	3	1.431	1.217	0.044
	Synbiotic	162	0	3	1	0	2	1.173	1.224	

**Table 4:** Comparison of bacteriological results between the group of patients using synbiotics and the group not using synbiotics.

Variable (Bacteriological Result)	Without Synbiotic *n* = 231 (100%) ^1^	Synbiotic *n* = 194 (100%) ^1^	*p* ^2^
Swab 1: *Acinetobacter lwoffii*	1 (0%)	1 (1%)	>0.99
Swab 1: *Aspergillus spp*.	1 (0%)	1 (1%)	>0.99
Swab 1: *Bacteroides ovatus*	1 (0%)	1(1%)	>0.99
Swab 1: *Candida spp.*	1 (0%)	2 (1%)	0.88
Swab 1: *Corynebacterium acconlens*	1 (0%)	0 (0%)	>0.99
Swab 1: *Citrobacter freundii*	4 (2%)	7 (4%)	0.36
Swab 1: *Citrobacter koseri*	5 (2%)	7(4%)	0.55
Swab 1: *Enterobacter aerogenes*	3 (1%)	4 (2%)	0.82
Swab 1: *Enterobacter cloacae*	6 (3%)	3 (2%)	0.68
Swab 1: *Enterococcus faecalis*	2 (1%)	3 (2%)	0.84
Swab 1: *Escherichia coli*	37 (16%)	20 (10%)	0.11
Swab 1: physiological flora	1 (0%)	6 (3%)	0.078
Swab 1: *Haemophilus influenzae*	5 (2%)	0 (0%)	0.11
Swab 1: *Hafnia alvei*	0 (0%)	1 (1%)	0.93
Swab 1: *Klebsiella aerogenes*	1 (0%)	0 (0%)	>0.99
Swab 1: *Klebsiella oxytoca*	17 (7%)	18 (9%)	0.59
Swab 1: *Klebsiella pneumoniae*	12 (5%)	19 (10%)	0.10
Swab 1: *Morganella morganii*	4 (2%)	5 (3%)	0.79
Swab 1: *Prevotella oralis*	1 (0%)	0 (0)%	>0.99
Swab 1: *Proteus hauseri*	1 (0%)	0 (0%)	>0.99
Swab 1: *Proteus mirabilis*	9 (4%)	9 (5%)	0.89
Swab 1: *Pseudomonas aeruginosa*	4 (2%)	5 (3%)	0.79
Swab 1: *Raoultella planticola*	2 (1%)	2 (1%)	>0.99
Swab 1: *Serratia liquefaciens*	1 (0%)	0 (0%)	>0.99
Swab 1: *Serratia marcescens*	4 (2%)	1 (1%)	0.48
Swab 1: *Staphylococcus aureus*	59 (26%)	59 (30%)	0.31
Swab 1: *Staphylococcus aureus MSSA*	11 (5%)	2 (1%)	0.052
Swab 1: *Staphylococcus epidermidis*	11 (5%)	2 (1%)	0.12
Swab 1: *Staphylococcus hominis*	1 (0%)	1 (1%)	>0.99
Swab 1: *Staphylococcus lugdunensis*	1 (0%)	0 (0%)	>0.99
Swab 1: *Coagulase-negative Staphylococcus spp.*	14 (6%)	0 (0%)	0.001
Swab 1: *Stenotrophomonas maltophilia*	1 (0%)	0 (0%)	>0.99
Swab 1: *Streptococcus agalactiae*	2 (1%)	0 (0%)	0.56
Swab 1: *Group B beta-hemolytic Streptococcus*	1 (0%)	0 (0%)	>0.99
Swab 1: *Streptococcus canis*	1 (0%)	0 (0%)	>0.99
Swab 1: *Streptococcus viridans*	1 (0%)	0 (0%)	>0.99
Swab 1: *Streptococcus pneumoniae*	3 (1%)	0 (0%)	0.31
Swab 1: *Streptococcus pyogenes*	1 (0%)	1 (1%)	>0.99
Swab 1: negative	50 (22%)	44 (23%)	0.89
Swab 1: *saprophytic cocci*	0 (0%)	1 (1%)	0.93
Swab 2: *Acinetobacter baumannii*	3 (1%)	2 (1%)	>0.99
Swab 2: *Acinetobacter lwoffii*	0 (0%)	1 (1%)	0.93
Swab 2: *Acinetobacter ursingii*	1 (0%)	0 (0%)	>0.99
Swab 2: *Candida spp.*	1 (0%)	0 (0%)	>0.99
Swab 2: *Citrobacter freundii*	1 (0%)	3 (2%)	0.50
Swab 2: *Citrobacter koseri*	3 (1%)	3 (2%)	>0.99
Swab 2: *Citrobakter braakii*	1 (0%)	0 (0%)	>0.99
Swab 2: *Corynebacterium accolens*	1 (0%)	0 (0%)	>0.99
Swab 2: *Corynebacterium pseudodiphtheriticum*	3 (1%)	0 (0%)	0.31
Swab 2: *Enterobacter cloacae*	2 (1%)	5 (3%)	0.32
Swab 2: *Enterococcus faecalis*	1 (0%)	0 (0%)	>0.99
Swab 2: *Escherichia coli*	15 (6%)	8 (4%)	0.39
Swab 2: physiological flora	16 (7%)	22 (11%)	0.16
Swab 2: *Haemophilus influenzae*	5 (2%)	1 (1%)	0.31
Swab 2: *Klebsiella oxytoca*	4 (2%)	9 (5%)	0.15
Swab 2: *Klebsiella pneumoniae*	7 (3%)	6 (3%)	>0.99
Swab 2: *Moraxella catarhalis*	0 (0%)	1 (1%)	0.93
Swab 2: *Moraxella nonliquefaciens*	1 (0%)	0 (0%)	>0.99
Swab 2: *Morganella morganii*	1 (0%)	0 (0%)	>0.99
Swab 2: *Pantoea spp.*	2 (1%)	0 (0%)	0.56
Swab 2: *Proteus mirabilis*	7 (3%)	2 (1%)	0.28
Swab 2: *Pseudomonas aeruginosa*	2 (1%)	4 (2%)	0.53
Swab 2: *Pseudomonas luteola*	0 (0%)	1 (1%)	0.93
Swab 2: *Raoultella ornithinolytica*	1 (0%)	0 (0%)	>0.99
Swab 2: *Raoultella planticola*	1 (0%)	0 (0%)	>0.99
Swab 2: *Serratia marcescens*	4 (2%)	2 (1%)	0.84
Swab 2: *Staphylococcus aureus*	50 (22%)	53 (27%)	0.18
Swab 2: *Staphylococcus aureus MSSA*	12 (5%)	3 (2%)	0.077
Swab 2: *Coagulase-negative Staphylococcus spp*.	24 (10%)	0 (0%)	<0.001
Swab 2: *Streptococcus constellatus*	1 (0%)	0 (0%)	>0.99
Swab 2: *Streptococcus viridans*	1 (0%)	0 (0%)	>0.99
Swab 2: *Streptococcus pneumoniae*	6 (3%)	3 (2%)	0.68
Swab 2: negative	41 (18%)	46 (24%)	0.16

^1^ n (%); ^2^ Pearson’s chi-squared test; Swab 1—pre-ESS swab, Swab 2—post-ESS swab. In the laboratory, a slight growth of *Staphylococcus epidermidis* was classified as physiological flora.

**Table 5:** Comparison of bacteriological results between groups, limited to patients who were not treated with antibiotics.

Variable (Bacteriological Result)	Without Synbiotic *n* = 78 ^1^	Synbiotic *n* = 107 ^1^	*p* ^2^
Swab 1: *Candida albicans*	1 (1%)	2 (2%)	>0.99
Swab 1: *Citrobacter freundii*	0 (0%)	5 (5%)	0.14
Swab 1: *Citrobacter koseri*	1 (1%)	3 (3%)	0.85
Swab 1: *Enterobacter aerogenes*	1 (1%)	2 (2%)	>0.99
Swab 1: *Enterobacter cloacae*	2 (3%)	2 (2%)	>0.99
Swab 1: *Enterococcus faecalis*	0 (0%)	2 (2%)	0.62
Swab 1: *Escherichia coli*	9 (12%)	2 (2%)	0.015
Swab 1: physiological flora	1 (1%)	2 (2%)	>0.99
Swab 1: *Hafnia alvei*	0 (0%)	1 (1%)	>0.99
Swab 1: *Klebsiella oxytoca*	4 (5%)	8 (7%)	0.74
Swab 1: *Klebsiella pneumoniae*	2 (3%)	8 (7%)	0.26
Swab 1: *Morganella morganii*	1 (1%)	3 (3%)	0.85
Swab 1: *Prevotella oralis*	1 (1%)	0 (0%)	0.87
Swab 1: *Proteus mirabilis*	3 (4%)	3 (3%)	>0.99
Swab 1: *Pseudomonas aeruginosa*	1 (1%)	4 (4%)	0.58
Swab 1: *Raoultella planticola*	0 (0%)	1 (1%)	>0.99
Swab 1: *Serratia marcescens*	0 (0%)	1 (1%)	>0.99
Swab 1: *Staphylococcus aureus*	9 (12%)	34 (32%)	0.002
Swab 1: *Staphylococcus aureus MSSA*	6 (8%)	1 (1%)	0.047
Swab 1: *Staphylococcus epidermidis*	5 (6%)	1 (1%)	0.40
Swab 1: *Staphylococcus lugdunensis*	1 (1%)	0 (0%)	0.87
Swab 1: *Stenotrophomonas maltophilia*	1 (1%)	0 (0%)	0.87
Swab 1: *Streptococcus pyogenes*	1 (1%)	1 (1%)	>0.99
Swab 1: negative	34 (44%)	35 (33%)	0.17
Swab 1: *saprophytic cocci*	0 (0%)	1 (1%)	>0.99
Swab 2: *Acinetobacter baumannii*	0 (0%)	1 (1%)	>0.99
Swab 2: *Acinetobacter lwoffii*	0 (0%)	1 (1%)	>0.99
Swab 2: *Citrobacter freundii*	1 (1%)	3 (3%)	0.85
Swab 2: *Citrobacter koseri*	1 (1%)	3 (3%)	0.85
Swab 2: *Enterobacter cloacae*	2 (3%)	4 (4%)	0.98
Swab 2: *Escherichia coli*	3 (4%)	2 (2%)	0.72
Swab 2: physiological flora	10 (13%)	14 (13%)	>0.99
Swab 2: *Haemophilus influenzae*	2 (3%)	1 (1%)	0.78
Swab 2: *Klebsiella oxytoca*	1 (1%)	6 (6%)	0.26
Swab 2: *Klebsiella pneumoniae*	1 (1%)	6 (6%)	0.26
Swab 2: *Moraxella catarhalis*	0 (0%)	1 (1%)	>0.99
Swab 2: *Pantoea spp.*	1 (1%)	0 (0%)	0.87
Swab 2: *Proteus mirabilis*	1 (1%)	2 (2%)	>0.99
Swab 2: *Pseudomonas aeruginosa*	0 (0%)	3 (3%)	0.37
Swab 2: *Raoultella planticola*	1 (1%)	0 (0%)	0.87
Swab 2: *Serratia marcescens*	1 (1%)	2 (2%)	>0.99
Swab 2: *Staphylococcus aureus*	19 (24%)	33 (31%)	0.42
Swab 2: *Staphylococcus aureus MSSA*	8 (10%)	2 (2%)	0.17
Swab 2: *Streptococcus pneumoniae*	0 (0%)	2 (2%)	0.62
Swab 2: negative	25 (32%)	25 (23%)	0.25

^1^ n (%); ^2^ Pearson’s chi-squared test.

**Table 6:** Comparison of bacteriological results between groups before and after the ESS procedure.

	Without Synbiotic			Synbiotic		
Variable (Bacteriological Result)	Before *n* = 231 ^1^	After *n* = 231 ^1^	*p* ^2^	Before *n* = 194 ^1^	After *n* = 194 ^1^	*p* ^2^
*Acinetobacter baumannii*	0 (0%)	3 (1%)	0.25	0 (0%)	2 (1%)	0.48
*Acinetobacter lwoffii*	1 (0%)	0 (0%)	>0.99	0 (0%)	1 (1%)	>0.99
*Acinetobacter ursingii*	0 (0%)	1 (0%)	>0.99	0 (0%)	0 (0%)	
*Aspergillus spp.*	1 (0%)	0 (0%)	>0.99	0 (0%)	0 (0%)	
*Bacteroides ovatus*	1 (0%)	0 (0%)	>0.99	0 (0%)	0 (0%)	
*Candida albicans*	1 (0%)	0 (0%)	>0.99	2 (1%)	0 (0%)	0.48
*Candida spp.*	0 (0%)	1 (0%)	>0.99	0 (0%)	0 (0%)	
*Corynebacterium acconlens*	1 (0%)	0 (0%)	>0.99	0 (0%)	0 (0%)	
*Citrobacter freundii*	4 (2%)	1 (0%)	0.37	7 (4%)	3 (2%)	0.34
*Citrobacter koseri*	5 (2%)	3 (1%)	0.72	7 (4%)	3 (2%)	0.34
*Citrobakter brakii*	0 (0%)	1 (0%)	>0.99	0 (0%)	0 (0%)	
*Corynebacterium accolens*	0 (0%)	1 (0%)	>0.99	0 (0%)	0 (0%)	
*Corynebacterium pseudodiphtheriticum*	0 (0%)	3 (1%)	0.25	0 (0%)	0 (0%)	
*Enterobacter aerogenes*	3 (1%)	0 (0%)	0.25	4 (2%)	0 (0%)	0.13
*Enterobacter cloacae*	6 (3%)	2 (1%)	0.28	3 (2%)	5 (3%)	0.72
*Enterococcus faecalis*	2 (1%)	1 (0%)	>0.99	3 (2%)	0 (0%)	0.25
*Escherichia coli*	37 (16%)	15 (6%)	0.002	20 (10%)	8 (4%)	0.031
Physiological flora	1 (0%)	16 (7%)	<0.001	6 (3%)	22 (11%)	0.003
*Haemophilus influenzae*	5 (2%)	5 (2%)	>0.99	0 (0%)	1 (1%)	>0.99
*Hafnia alvei*	0 (0%)	0 (0%)		1 (1%)	0 (0%)	>0.99
*Klebsiella aerogenes*	1 (0%)	0 (0%)	>0.99	0 (0%)	0 (0%)	
*Klebsiella oxytoca*	17 (7%)	4 (2%)	0.007	18 (9%)	9 (5%)	0.11
*Klebsiella pneumoniae*	12 (5%)	7 (3%)	0.35	19 (10%)	6 (3%)	0.013
*Moraxella catarhalis*	0 (0%)	0 (0%)		0 (0%)	1 (1%)	>0.99
*Moraxella nonliquefaciens*	0 (0%)	1 (0%)	>0.99	0 (0%)	0 (0%)	
*Morganella morganii*	4 (2%)	1 (0%)	0.37	5 (3%)	0 (0%)	0.072
*Pantoea spp.*	0 (0%)	2 (1%)	0.48	0 (0%)	0 (0%)	
*Prevotella oralis*	1 (0%)	0 (0%)	>0.99	0 (0%)	0 (0%)	
*Proteus hauseri*	1 (0%)	0 (0%)	>0.99	0 (0%)	0 (0%)	
*Proteus mirabilis*	9 (4%)	7 (3%)	0.80	9 (5%)	2 (1%)	0.066
*Pseudomonas aeruginosa*	4 (2%)	2 (1%)	0.68	5 (3%)	4 (2%)	>0.99
*Pseudomonas luteola*	0 (0%)	0 (0%)		0 (0%)	1 (1%)	>0.99
*Raoultella ornithinolytica*	0 (0%)	1 (0%)	>0.99	0 (0%)	0 (0%)	
*Raoultella planticola*	2 (1%)	1 (0%)	>0.99	2 (1%)	0 (0%)	0.48
*Serratia liquefaciens*	1 (0%)	0 (0%)	>0.99	0 (0%)	0 (0%)	
*Serratia marcescens*	4 (2%)	4 (2%)	>0.99	1 (1%)	2 (1%)	>0.99
*Staphylococcus aureus*	59 (26%)	50 (22%)	0.38	59 (30%)	53 (27%)	0.58
*Staphylococcus aureus MSSA*	11 (5%)	12 (5%)	>0.99	2 (1%)	3 (2%)	>0.99
*Staphylococcus epidermidis*	11 (5%)	0 (0%)	0.002	2 (1%)	0 (0%)	0.48
*Staphylococcus hominis*	1 (0%)	0 (0%)	>0.99	1 (1%)	0 (0%)	>0.99
*Staphylococcus lugdunensis*	1 (0%)	0 (0%)	>0.99	0 (0%)	0 (0%)	
*Coagulase-negative Staphylococcus spp.*	14 (6%)	24 (10%)	0.13	0 (0%)	0 (0%)	
*Stenotrophomonas maltophilia*	1 (0%)	0 (0%)	>0.99	0 (0%)	0 (0%)	
*Streptococcus constellatus*	0 (0%)	1 (0%)	>0.99	0 (0%)	0 (0%)	
*Streptococcus agalactiae*	2 (1%)	0 (0%)	0.48	0 (0%)	0 (0%)	
*Group B beta-hemolytic Streptococcus*	1 (0%)	0 (0%)	>0.99	0 (0%)	0 (0%)	
*Streptococcus canis*	1 (0%)	0 (0%)	>0.99	0 (0%)	0 (0%)	
*Streptococcus viridans*	1 (0%)	1 (0%)	>0.99	0 (0%)	0 (0%)	
*Streptococcus pneumoniae*	3 (1%)	6 (3%)	0.50	0 (0%)	3 (2%)	0.25
*Streptococcus pyogenes*	1 (0%)	0 (0%)	>0.99	1 (1%)	0 (0%)	>0.99
Negative swab	50 (22%)	41 (18%)	0.35	44 (23%)	46 (24%)	0.90
*Saprophytic cocci*	0 (0%)	0 (0%)		1 (1%)	0 (0%)	>0.99
Type of bacteria			0.001			0.038
Gram-positive bacteria	73 (41%)	77 (62%)		49 (35%)	48 (52%)	
Gram-negative bacteria	75 (43%)	31 (25%)		74 (53%)	35 (38%)	
Gram-positive and Gram-negative bacteria	28 (16%)	16 (13%)		17 (12%)	10 (11%)	

^1^ n (%); ^2^ Pearson’s chi-squared test.

**Table 7:** Comparison of bacteriological results between groups before and after the ESS procedure only in patients with confirmed absence of antibiotic use.

	Without Synbiotic			Synbiotic		
Variable (Bacteriological Result)	Before *n* = 78 ^1^	After *n* = 78 ^1^	*p* ^2^	Before *n* = 107 ^1^	After *n* = 107 ^1^	*p* ^2^
*Acinetobacter baumannii*	0 (0%)	0 (0%)		0 (0%)	1 (1%)	>0.99
*Acinetobacter lwoffii*	0 (0%)	0 (0%)		0 (0%)	1 (1%)	>0.99
*Candida albicans*	1 (1%)	0 (0%)	>0.99	2 (2%)	0 (0%)	0.48
*Citrobacter freundii*	0 (0%)	1 (1%)	>0.99	5 (5%)	3 (3%)	0.72
*Citrobacter koseri*	1 (1%)	1 (1%)	>0.99	3 (3%)	3 (3%)	>0.99
*Enterobacter aerogenes*	1 (1%)	0 (0%)	>0.99	2 (2%)	0 (0%)	0.48
*Enterobacter cloacae*	2 (3%)	2 (3%)	>0.99	2 (2%)	4 (4%)	0.68
*Enterococcus faecalis*	0 (0%)	0 (0%)		2 (2%)	0 (0%)	0.48
*Escherichia coli*	9 (12%)	3 (4%)	0.13	2 (2%)	2 (2%)	>0.99
Physiological flora	1 (1%)	10 (13%)	0.012	2 (2%)	14 (13%)	0.004
*Haemophilus influenzae*	0 (0%)	2 (3%)	0.48	0 (0%)	1 (1%)	>0.99
*Hafnia alvei*	0 (0%)	0 (0%)		1 (1%)	0 (0%)	>0.99
*Klebsiella oxytoca*	4 (5%)	1 (1%)	0.36	8 (7%)	6 (6%)	0.78
*Klebsiella pneumoniae*	2 (3%)	1 (1%)	>0.99	8 (7%)	6 (6%)	0.78
*Moraxella catarhalis*	0 (0%)	0 (0%)		0 (0%)	1 (1%)	>0.99
*Morganella morganii*	1 (1%)	0 (0%)	>0.99	3 (3%)	0 (0%)	0.24
*Pantoea spp.*	0 (0%)	1 (1%)	>0.99	0 (0%)	0 (0%)	
*Prevotella oralis*	1 (1%)	0 (0%)	>0.99	0 (0%)	0 (0%)	
*Proteus mirabilis*	3 (4%)	1 (1%)	0.61	3 (3%)	2 (2%)	>0.99
*Pseudomonas aeruginosa*	1 (1%)	0 (0%)	>0.99	4 (4%)	3 (3%)	>0.99
*Raoultella planticola*	0 (0%)	1 (1%)	>0.99	1 (1%)	0 (0%)	>0.99
*Serratia marcescens*	0 (0%)	1 (1%)	>0.99	1 (1%)	2 (2%)	>0.99
*Staphylococcus aureus*	9 (12%)	19 (24%)	0.060	34 (32%)	33 (31%)	>0.99
*Staphylococcus aureus MSSA*	6 (8%)	8 (10%)	0.78	1 (1%)	2 (2%)	>0.99
*Staphylococcus epidermidis*	5 (6%)	0 (0%)	0.069	1 (1%)	0 (0%)	>0.99
*Staphylococcus lugdunensis*	1 (1%)	0 (0%)	>0.99	0 (0%)	0 (0%)	
*Stenotrophomonas maltophilia*	1 (1%)	0 (0%)	>0.99	0 (0%)	0 (0%)	
*Streptococcus pneumoniae*	0 (0%)	0 (0%)		0 (0%)	2 (2%)	0.48
*Streptococcus pyogenes*	1 (1%)	0 (0%)	>0.99	1 (1%)	0 (0%)	>0.99
Negative swab	34 (44%)	25 (32%)	0.19	35 (33%)	25 (23%)	0.17
*saprophytic cocci*	0 (0%)	0 (0%)		1 (1%)	0 (0%)	>0.99
Type of bacteria			0.16			0.70
Gram-positive bacteria	18 (43%)	23 (62%)		28 (42%)	30 (48%)	
Gram-negative bacteria	20 (48%)	10 (27%)		29 (44%)	26 (42%)	
Gram-positive and Gram-negative bacteria	4 (10%)	4 (11%)		9 (14%)	6 (10%)	

^1^ n (%); ^2^ Pearson’s chi-squared test.

## Data Availability

The original contributions presented in this study are included in the article. Further inquiries can be directed to the corresponding author(s).

## Supplementary Materials

The following supporting information can be downloaded at: https://www.mdpi.com/article/10.3390/medicina61071306/s1, STROBE Checklist.

## Abbreviations

The following abbreviations are used in this manuscript:

CRS	Chronic Rhinosinusitis
AR	Allergic Rhinitis
ESS	Endoscopic Sinus Surgery
EPOS 2020	European Position Paper on Rhinosinusitis and Nasal Polyps 2020
MRSA	Methicillin-Resistant Staphylococcus aureus
MSSA	Methicillin-Sensitive Staphylococcus aureus
NSAID	Non-Steroidal Anti-Inflammatory Drug
CRSwNP	Chronic Rhinosinusitis with Nasal Polyps
CRSsNP	Chronic Rhinosinusitis without Nasal Polyps
VAS	Visual Analog Scale
SNOT-22	Sino-Nasal Outcome Test-22
